# Nutrition surveillance using a small open cohort: experience from Burkina Faso

**DOI:** 10.1186/s12982-016-0052-0

**Published:** 2016-11-15

**Authors:** Mathias Altmann, Christophe Fermanian, Boshen Jiao, Chiara Altare, Martin Loada, Mark Myatt

**Affiliations:** 1 Action Contre la Faim, 16 Boulevard Douaumont, 75017 Paris, France; 2 EHESP Sorbonne Paris Cité, Avenue du Professeur Léon-Bernard, 35043 Rennes, France; 3 Ecole des Hautes Etudes en Santé Publique, Avenue du Professeur Léon-Bernard, 35043 Rennes, France; 4 Action Contre la Faim, Ouagadougou, Burkina Faso; 5 Brixton Heath, Llawryglyn, Powys, Wales UK

**Keywords:** Nutrition, Surveillance, Burkina Faso, Selection bias, Observational bias, Humanitarian

## Abstract

**Background:**

Nutritional surveillance remains generally weak and early warning systems are needed in areas with high burden of acute under-nutrition. In order to enhance insight into nutritional surveillance, a community-based sentinel sites approach, known as the Listening Posts (LP) Project, was piloted in Burkina Faso by Action Contre la Faim (ACF). This paper presents ACF’s experience with the LP approach and investigates potential selection and observational biases.

**Methods:**

Six primary sampling units (PSUs) were selected in each livelihood zone using the centric systematic area sampling methodology. In each PSU, 22 children aged between 6 and 24 months were selected by proximity sampling. The prevalence of GAM for each month from January 2011 to December 2013 was estimated using a Bayesian normal–normal conjugate analysis followed by PROBIT estimation. To validate the LP approach in detecting changes over time, the time trends of MUAC from LP and from five cross-sectional surveys were modelled using polynomial regression and compared by using a Wald test. The differences between prevalence estimates from the two data sources were used to assess selection and observational biases.

**Results:**

The 95 % credible interval around GAM prevalence estimates using LP approach ranged between +6.5 %/−6.0 % on a prevalence of 36.1 % and +3.5 %/−2.9 % on a prevalence of 10.8 %. LP and cross-sectional surveys time trend models were well correlated (p = 0.6337). Although LP showed a slight but significant trend for GAM to decrease over time at a rate of −0.26 %/visit, the prevalence estimates from the two data sources showed good agreement over a 3-year period.

**Conclusions:**

The LP methodology has proved to be valid in following trends of GAM prevalence for a period of 3 years without selection bias. However, a slight observational bias was observed, requiring a periodical reselection of the sentinel sites. This kind of surveillance project is suited to use in areas with high burden of acute under-nutrition where early warning systems are strongly needed. Advocacy is necessary to develop sustainable nutrition surveillance system and to support the use of surveillance data in guiding nutritional programs.

## Background

*Nutrition surveillance* means “to watch over nutrition in order to make decisions that lead to improvements in nutrition in populations” [1]. Nutrition surveillance refers to a continuous process and focuses on monitoring trends over time, rather than providing one-time estimates of (e.g.) absolute levels of the prevalence of malnutrition, in order to identify and respond to crises in a timely manner [2].

Although they have been recognized as an important component in fighting malnutrition, nutritional surveillance systems remain weak in most developing countries [3]. Reasons for this include (1) no common agreement on the best methods to implement nutrition surveillance, (2) a lack of confidence in surveillance data, and (3) little comparable data on the costs of different potentially effective systems that would justify investments in such a system [2, 4]. It is, therefore, essential for practitioners to share experiences regarding nutritional surveillance in order to provide insights into what works and what does not work in the field.

Nutritional surveillance data tend to come from two main sources: administrative (e.g. health facility/feeding centre caseloads and schools health services reports) and repeated probability sample household surveys [2, 5]. Limitations of administrative data are well known. There may be a selection bias due to incomplete distribution of facilities and populations that are covered by programs contributing data [6]. Even when the facilities are well running, only people with better access may attend clinics or nutrition program sites, thus underestimating the true prevalence/incidence of the condition of interest. Furthermore, unless active case-finding is used, beneficiaries may tend to come to the facilities only when the disease is severe. This means that indicators may lag behind incidence, making surveillance data inappropriate for an early warning system. The second data source, repeated probability sample household surveys, is the most commonly used approach to nutrition surveillance [5, 7, 8]. Surveys provide a representative picture of the situation at a given time and allow comparisons over time and between geographical areas. However, unless they are repeated frequently enough, surveys may miss seasonal effect and cannot provide timely information on changes over time [9].

Less attention has been given to the community-based sentinel sites approach to nutrition surveillance. These surveillance systems are characterised by the selection of a small sample of communities from which a set of information is collected regularly. There are two main criticisms to the sentinel approach. First, the purposive sampling of selected sites according to predefined criteria (e.g. the most “vulnerable” settlements) results into non-representative estimates (likely overestimates) [4]. Second, an observational effect that acts to reduce prevalence over time as the selected sites tend to be progressively positively affected by the inputs of the survey teams (e.g. giving education, advice and counselling, referral of cases for treatment, and treating illness) [10]. It is not clear, however, that a statistically representative sample, as might be used in a population survey, is an essential attribute of a surveillance system. It may, for example, be more useful to select and watch over communities that are vulnerable to shocks so as to detect potential crises early in their development. Experiences of sentinel sites nutrition surveillance have been reported from Sudan [11] and the Central African Republic [12].

This paper presents the experience of the international non-governmental organization Action Contre la Faim (ACF) with nutrition surveillance using a community-based sentinel sites approach, known as the Listening Posts (LP) project. We established a surveillance system in order to estimate nutritional and food security needs and to identify when and where these needs were highest. The system was set up to describe patterns over time, and also to provide accurate estimates of the point prevalence of acute undernutrition and to provide predictions of the caseloads. In this paper, we report and examine our experience in Burkina Faso in order to assess the reliability and validity of the LP method compared to repeated cross-sectional surveys in terms of selection and observation biases.

## Methods

### Selection of livelihood zones

Criteria for selecting the setting were as follow: existence of a programme implemented by ACF; availability of sufficient capacity to conduct surveillance; nationally and locally weak nutrition information systems in the government sector; no other sentinel surveillance system in place in the government sector; involvement of the government in the selection of the livelihood zone (LHZ); and “vulnerability” of LHZ on the basis of an Household Economy Approach (HEA) food security assessment [13]. The LHZ was defined as a geographical area where people share broadly the same patterns of access to food and income, and have the same access to local markets.

**Fig. 1:**
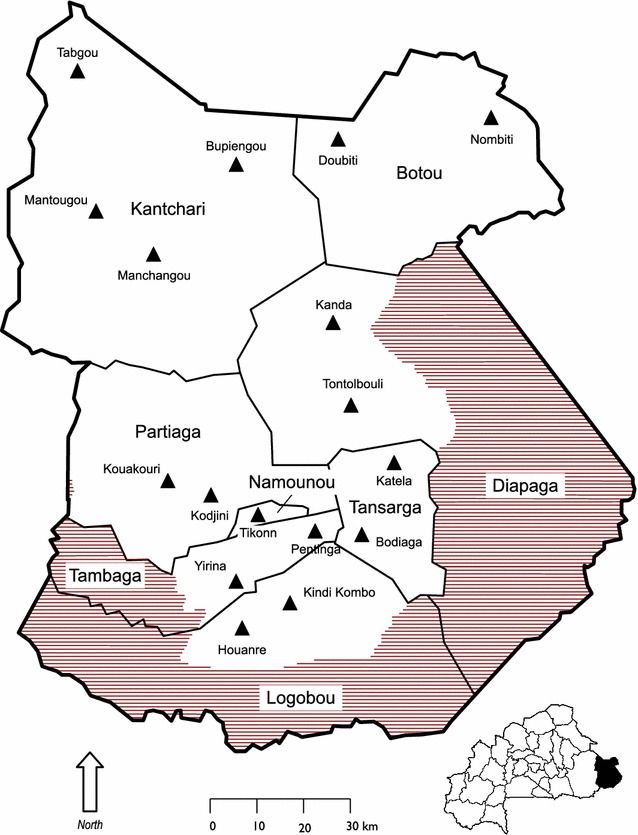
Zones selected for surveillance, Tapoa province, Burkina Faso. Sentinel sites are shown with *filled triangle*

Based on our selection criteria, we piloted our methodology in Tapoa province (Burkina Faso). In 2011, Tapoa province had a population of about 400,000 people, 17.4 % of which were children under the age of five [14]. Prevalence of Global Acute Malnutrition (GAM) (defined as weight-for-height Z-score <−2) was estimated to be 12.3 % (9.5–15.9 %) in children aged between 6 and 59 months [15]. This is one of the highest GAM prevalence in the country. Surveillance started in January 2011 in 3 LHZ (Fig. 1): (1) agro-pastoral (north) (2) subsistence farming (centre) (3) cash farming and hunting (south).

### Sample size calculation

The sample size was calculated taking both accuracy and costs into account, keeping in mind that low cost is an important factor for the sustainability of surveillance systems. The following aspects were included in the calculation of the sample size: (1) age range was reduced to 6–24 months. Besides the fact that this reduced the size of the universe (small population), this age group is most vulnerable to acute malnutrition; (2) semi-longitudinal design: the use of an “open cohort” (see “top-up replacement and referral” below) decreased the required sample size through reduction of between round sampling variation compared to taking a new sample at each round; (3) estimation of GAM was done using a Bayesian normal–normal conjugate analysis with an objective prior followed by a PROBIT analysis [16–19]. The Bayesian conjugate analysis was used because the prior contains information that contributes “pseudo-observations” to the conjugate analysis. This means that the Bayesian conjugate analysis will have a larger effective sample size than a frequentist analysis of similar sample size provided that there is no gross conflict between the prior and the likelihood (i.e. observed) data. A larger effective sample size translates to improved precision of estimates [19, 20]. The Bayesian normal–normal conjugate analysis yields posterior estimates of the mean and standard deviation (SD) which are the inputs required by the inverse cumulative distribution function used in the PROBIT estimator. The PROBIT estimator retains information about scale and variability that is lost by the classical approach when the data are coded to a case/not case binary variable. This retained information allows the PROBIT approach to return estimates with improved precision compared to the classical (i.e. case counting) approach [17, 18], making the method well suited to work with small samples. With these conditions, and using computer-based simulations using data derived from cross-sectional surveys, we calculated that a sample size of *n* = 96 from each LHZ could be expected to yield a 95 % credible interval (CI) of ±10 % or better at any level of prevalence. A sample size of *n* = 132 children was selected in order to ensure that useful precision is achieved.

### Sampling and eligibility criteria

A two-stage cluster sample of children was taken from each selected LHZ. Six primary sampling units (PSUs), also called “Listening Posts”, were selected using the centric systematic area sampling (CSAS) methodology, by which the sample selected was reasonably evenly distributed across the survey area. This type of sample provides implicit stratification by spreading the sample properly among sub-groups of the population such as rural, urban, peri-urban populations, administrative areas, ethnic sub-populations, religious sub-populations, and socio-economic groups [21–25]. This tends to improve precision of survey estimates from survey data. In the second sampling stage, we selected 22 children from each Listening Post (PSUs) using the Expanded Program on Immunization (EPI) household sampling scheme: the first household was selected by choosing a random direction from the centre of the community, counting the houses along that route, and picking one at random, and the sampling was continued by choosing the household nearest to the preceding one that included an eligible child [26]. All children aged between 6 and 24 months in selected households were included in the sample. Since a child falling into such a narrow age range would not be found in every household, the sample was spread widely across the PSU community [26]. This procedure provided the same advantage as implicit stratification by ensuring that all parts of the PSU were sampled.

### Top-up replacement and referral

When a child reached his or her second birthday, they were replaced by another child aged between 6 and 9 months not already in the cohort (the “top-up sample”) from the nearest household with an eligible child. Before being replaced, nutritional measurements were done and the survey questionnaire administered. A dead or lost-to-follow up (e.g. moved away) child was replaced by another child not already in the cohort and of similar age from the nearest household with an eligible child. These procedures ensured that the age structure of the cohort remained constant between surveillance rounds so that prevalence estimates would not be influenced by aging of the surveillance cohort. All children with a mid-upper arm circumference (MUAC) below 125 mm, as well as sick children, were referred to the nearest health centre.

### Data collection

Two interviewers for the three LHZ were trained to perform the sampling protocol, the required anthropometric measurements, and apply the survey questionnaire. Regular supervisions were conducted to ensure anthropometric measurements were done correctly. Each interviewer visited one Listening Post (PSU) per day, and performed interviews of mothers and measurements (weight and MUAC) of 22 children and top-up sampling when this was required. Data were collected during the first 2 weeks of each month. In order to avoid an interviewer bias, monthly rotations were organized in the visited LP between the two interviewers. 36 monthly rounds of data collection were performed and are included in the analysis presented here. Anthropometric measurement (weight and MUAC), morbidity (prevalence of diarrhoea in the last 15 days), infant and young child feeding (IYCF) practices, food security, and water, sanitation and hygiene (WASH) indicators were collected using a paper based questionnaire. In this article, we will concentrate on the prevalence of global acute malnutrition (GAM), defined as MUAC <125 mm, which is recognized as a sensible indicator to capture variations of the nutrition status at community level [27]. One supervisor prepared the planning of the interviewer, the questionnaires, checked for missing data and validated the data for analysis. Data was entered into an Excel spread sheet, together with quality assurance mechanisms such as cross-field consistency checks, legal value, and range checks.

### Data analysis

Design effect was calculated by dividing the standard error (SE) with clustering by the SE without clustering. For continuous variables, median and Inter-Quartile-Range (IQR) were calculated for the entire study period. The prevalence of GAM was estimated by MUAC with a case-defining threshold of 125 mm using a Bayesian normal–normal conjugate analysis followed by a PROBIT estimation approach. In the work reported, an objective prior was specified using the sex-combined median MUAC-for-age and the square of the sex-combined median negative z-score for children aged between 6 and 24 months taken from the WHO’s World Growth Standard (MGRS) reference population [28]. We used the population mean and variance parameter of the MGRS reference populations (i.e. 145 mm and 121 mm^2^, respectively). Using these parameters, the equation used to estimate the mean MUAC was:

1 $$\mu |survey\;data\sim\,N\left( {\frac{{145 + n\bar{x}}}{n + 1},\frac{121}{n + 1}} \right)$$

where *n* is the number of children in the sample and $\bar{x}$ is the mean MUAC observed in the survey.

We used these values (i.e. the posterior mean and variance) to find the 95 % credible interval for the posterior mean using the inverse cumulative normal distribution (NORMINV) function in Microsoft Excel. We translated the posterior mean and the associated 95 % credible interval into a proportion (i.e. prevalence) using the cumulative normal distribution (NORMDIST) function in Microsoft Excel using a threshold value of 125 mm. By using this function, we estimated the probability that a child selected at random from the monitored population would have a MUAC below 125 mm (which equates to prevalence).

Data were analysed by the program manager and validated by the ACF Department for Food and Livelihoods. For the purpose of surveillance, data were analysed on a monthly basis using means and estimated proportions of the different collected variables. When plotting the results as a time series, the effect of sampling variation was reduced by applying a simple low-pass filter (i.e. smoothing using a 3 months moving average).

### Comparison between LP and cross-sectional surveys

The external validity of the estimates from the LP survey was assessed by comparing the time trend and variability of LP data with that from other data sources. In Burkina Faso, available data included nutritional cross-sectional surveys using the Standardized Monitoring and Assessment of Relief and Transitions (SMART) methodology [29], conducted by ACF in Tapoa Province in March 2011, May 2012, December 2012, May 2013 and October 2013 (data not published but available upon request). In order to have the same age group as used for the LP surveillance system, only 6–24 months aged children were kept from the cross-sectional surveys.

First, the individual data of LP surveys from all the three LHZ were pooled. We modelled the time trends of continuous MUAC for both LP and cross-sectional data through forward stepwise curvilinear regression weighted by the population in each LHZ. Time (*T*), squared time (*T* ^2^) and cubed time (*T* ^3^) were entered into the model as independent variables. This led to the following polynomial model:

2 $$Y = \beta_{0} + \beta_{1} {\text{T}} + \beta_{2} {\text{T}}^{2} + \beta_{3} {\text{T}}^{3}$$

where *Y* is the continuous MUAC of the individuals assessed at time *T*, *β*_0_ is the intercept of the model, *β*_1_, *β*_2_ and *β*_3_ are the coefficients of respective terms *T*, *T* ^2^ and *T* ^3^.

Second, in order to compare estimates from the LP and cross-sectional surveys, we built a regression model by pooling data from both the LP and cross-sectional surveys sources and adding a new binary variable (*W*) with the value one when the corresponding data were from LP survey and zero when from cross-sectional survey. This led to the following model:

3 $$Y = \beta_{0}^{\prime } + \beta_{1}^{\prime } {\text{T}} + \beta_{2}^{\prime } {\text{T}}^{2} + \beta_{3}^{\prime } {\text{T}}^{3} + \beta_{4}^{\prime } {\text{W}}$$

where *Y* is the continuous MUAC of the individuals from both LP and cross-sectional data assessed at time *T*. $\beta_{0}^{\prime }$ is the intercept of model, $\beta_{1}^{\prime } ,\beta_{2}^{\prime }$ and $\beta_{3}^{\prime }$ are the coefficients of respective terms T, T^2^ and *T* ^3^, and *W* is the new variable mentioned above. Then we performed a Wald test to determine whether this new variable made a statistically significant improvement to the global regression model and to determine whether the coefficients in the LP and cross-sectional surveys models were significantly different from each other.

Third, we calculated the monthly mean MUAC for cross-sectional surveys and for LP separately, with pooled data weighted by the population of each LHZ. Using the Bayesian-PROBIT estimator, the prevalence of GAM was estimated for each data source, and the differences (D) in prevalence estimates between these two data sources plotted. We calculated the mean difference (${\bar{\text{D}}}$), the SD of the differences, and the estimated limits of agreement as ${\bar{\text{D}}}$ ± 1.96 SD. Agreement between the results from cross-sectional surveys and LP data was defined as being within these estimated limits of agreement [30]. To investigate the trend for differences in GAM prevalence as a function of time (observational bias), the associated least squares line was calculated using a simple regression of the form:

4 $$Y = \beta_{0}^{\prime \prime } + \beta_{1}^{\prime \prime } T^{\prime \prime }$$

where *Y* is the absolute differences (D) in prevalence estimates between LP and cross-sectional surveys assessed at time $T^{\prime \prime } ,\beta_{0}^{\prime \prime }$ is the intercept of regression line and $\beta_{1}^{\prime \prime }$ is the coefficient associated with time. The time at which differences would become significant was estimated as the point where this line crossed the upper confidence limit for the mean.

## Results

### Cohort profile

**Fig. 2:**
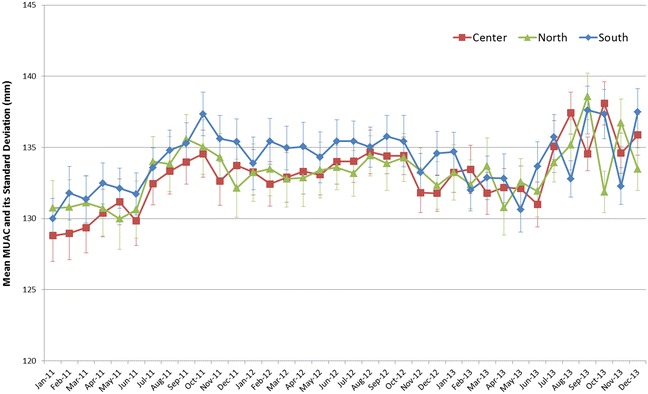
Mean MUAC and its standard deviation among 6–24 months aged children, in 3 Livelihood zones of Tapoa Province, Burkina Faso, 2011–2013

Sample sizes varied over the study period and between LHZ (Table 1). Overall, median of the mean age was 15.9 months and did not change over time and between LHZ. Overall, the SD around the mean MUAC had a median of 9.6 mm, without difference over time and between LHZ (Fig. 2).

**Table 1:** Characteristics of Listening Posts and cross-sectional surveys data sources, Tapoa Province, Burkina Faso, 2011–2013

Characteristics/livelihood zone	Nord	Centre	South	All 3 LHZ	Cross-sectional surveys (3 LHZ)
Number of rounds, n	36	36	36	36	5
Sample size (number of children) median (IQR)	119 (116–132)	139 (135–144)	162 (145–164)	418 (410–423)	287 (275–367)
Mean age (months) median (IQR)	15.6 (15.2–15.9)	16.0 (15.4–16.2)	16.0 (15.9–16.2)	15.9 (15.4–16.2)	14.7 (14.4–15.1)
Mean MUAC (mm) median (IQR)	133.4 (132.0–134.0)	133.3 (131.8–134.4)	134.6 (132.6–135.4)	133.8 (132.3–134.7)	133.2 (132.9–133.6)
SD (mm), median (IQR)	9.98 (9.42–10.60)	9.11 (8.66–9.67)	9.68 (9.01–10.23)	9.60 (9.19–10.04)	10.73 (10.43–10.77)
DEFF, median (IQR)	0.97 (0.76–1.31)	0.96 (0.80–1.25)	1.22 (1.07–1.46)	1.18 (1.09–1.31)	1.06 (1.04–1.14)

*DEFF* design effect, *IQR* inter quartile range, *LHZ* livelihood zone, *SD* standard deviation

Design effects were close to 1 over the study period and in all LHZs. The mean monthly replacement rate of the children in the cohort was 8.5 % (SD = 0.28 %) which is above the expected value due to aging of the cohort (1/18 = 5.6 %). The difference was explained by losses to follow up, defined as the absence of child at 3 consecutive visits before reaching their second birthday, which had a mean value of 2.8 % (SD = 0.17 %).

### Global acute malnutrition

**Fig. 3:**
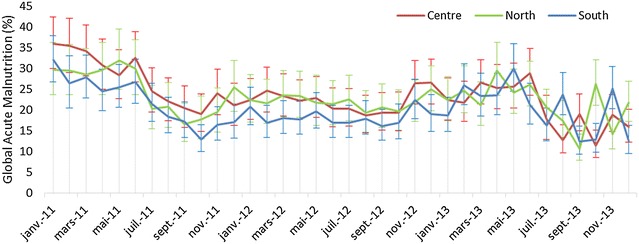
Prevalence (by MUAC) and its 95 % Credible Intervals of Global Acute Malnutrition among 6–24 months aged children, in 3 Livelihood zones of Tapoa Province, Burkina Faso, 2011–2013

95 % credible interval around GAM prevalence in a single LHZ ranged between +6.5 and −6.0 % on a prevalence of 36.1 % and between +3.5 and −2.9 % on a prevalence of 10.8 %. These levels of precision are consistent with the surveillance round having an effective sample size of *n* = 227 and *n* = 362 respectively. Using *n* = 132 proved therefore to be suitable for the LP sample design and analysis plan. The GAM prevalence estimates, based on mean MUAC, did not differ between the three LHZ’s (Fig. 3).

In the three LHZ of Tapoa province, GAM prevalence followed a seasonal variation with an increase during the dry season (January to May) and a decrease during the rainy season (June to September). Over the 3 years, GAM decreased during 2011, increased till the middle of 2013, and then decreased again during the last months of 2013 (Fig. 3).

### External validity

Characteristics of collected data in pooled LP and pooled cross-sectional surveys are displayed in Table 1. While mean age was slightly higher in LP than in cross-sectional surveys, mean MUAC was slightly lower in cross-sectional surveys. The SD was lower for LP than cross-sectional surveys, with a median of 9.60 and 10.73 mm, respectively. Design effect had a median of 1.18 and 1.06, for LP and cross-sectional surveys, respectively.

Continuous MUAC as the dependent variable was explained by time, squared time and cubed time as independent variables together (Table 2), which is consistent with the shape of time-series results plotted in Fig. 3. The Wald test results showed that the values of MUAC were not significantly different (p = 0.6337) between LP and cross-sectional surveys models. Thus the time trends of continuous MUAC were similar between the two models/data sources.

**Table 2:** Regression coefficients, confidence intervals and p value taking continuous MUAC as dependent variable, Tapoa province, Burkina Faso, 2011–2013

Variables	Coefficient	(95 % CI)	p value
LP model^a^			
Time	1.1597	(0.9897, 1.3296)	<0.001
Squared time	−0.0653	(−0.0757, −0.0549)	<0.001
Cubed time	0.0011	(0.0009, 0.0012)	<0.001
Intercept	128.02	(127.27, 128.76)	<0.001
Cross-sectional surveys model^b^			
Time	1.2149	(0.3633, 2.0661)	0.005
Squared time	−0.0773	(−0.1328, −0.0219)	0.006
Cubed time	0.0013	(0.0003, 0.0023)	0.008
Intercept	129.87	(127.14, 132.60)	<0.001

^a^Number of observations in LP model = 14,534

^b^Number of observations in cross-sectional surveys model = 1623

**Fig. 4:**
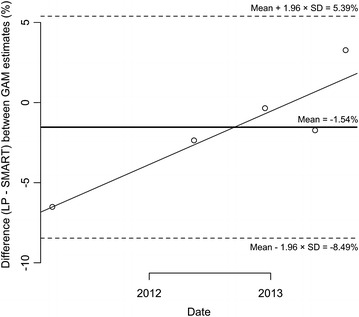
Difference in prevalence estimates between cross-sectional surveys and LP surveys, with mean and estimated 95 % limits of agreement, in 3 Livelihood zones of Tapoa province, Burkina Faso, 2011–2013

The monthly GAM was estimated with its 95 % credible intervals at five time points from cross-sectional surveys and LP surveys (Table 3). All differences (D) in prevalence estimates between cross-sectional surveys and LP lay within estimated confidence limits (−8.47 to 5.38 %) (Fig. 4), indicating fair agreement between the two datasets. However, the plot showed an increase as a function of time, namely as a function of the number of months; the corresponding equation was:

$${\text{y}} = 0.264045{\text{t}} - 6.744944$$

indicating a monthly increase of about 0.26 % in GAM prevalence for cross-sectional compared to LP.

**Table 3:** Prevalence (by mean MUAC) and its 95 % Credible Interval of Global Acute Malnutrition among 6–24 months aged children, by Listening Posts and cross-sectional surveys, Tapoa province, Burkina Faso, 2011–2013

Time points	Listening post surveys			Cross-sectional surveys		
	Mean MUAC (SD) in mm	Prevalence (%)	(95 % CI)	Mean MUAC (SD) in mm	Prevalence (%)	(95 % CI)
March 2011	130.6 (10.7)	30.12	(26.87, 33.52)	133.0 (11.7)	23.61	(20.21, 27.32)
May 2012	133.7 (10.2)	21.44	(18.69, 24.41)	134.8 (10.1)	19.08	(16.04, 22.46)
December 2012	132.9 (9.1)	23.01	(20.28, 26.12)	133.1 (10.7)	22.66	(19.11, 26.55)
May 2013	131.8 (9.6)	27.13	(24.03, 30.42)	132.2 (12.0)	25.40	(22.52, 28.47)
October 2013	135.8 (9.8)	18.41	(15.95, 21.08)	133.6 (10.4)	21.68	(18.80, 24.80)

Extrapolation of the line crossed the upper border of the confidence interval of the mean at time t = 45.96 months, indicating that the difference in GAM prevalence would probably become significant 3 years and 10 months after the beginning of the surveillance.

## Discussion

This paper presents the methodology for nutrition surveillance that was tested by ACF in a rural area of Burkina Faso, using a small open cohort in community-based sentinel sites. It provides detailed information on how to implement it and assess the reliability of its data in term of selection and observational biases. This methodology has been shown to be valid in following trends for a period of 3 years without selection bias.

Criticisms on sentinel system address the representativeness of the sample, including selection and observational biases. In our study, we used CSAS sampling rather than a purposive sampling technique. CSAS sampling has been shown to approximate random sampling [21] and is thereby representative of the survey area. Although mean age of the cohort remained constant over time, we cannot exclude that a selection bias might have been introduced when lost to follow up children were replaced at each round. Dataset comparisons between LP and repeated cross-sectional surveys did not detect any statistical difference in estimating mean MUAC over a 3-year period in our setting. Post-hoc power analysis showed that for these comparisons, the working power was between 79 and 97 %, thus the corresponding tests were not underpowered. Moreover, the use of the *Bayesian*-*PROBIT* approach to estimate GAM has increased the precision of prevalence estimate compared to the use of a frequentist analysis and classical estimator. Using a reduced age-group (i.e. <2 years old) proved to be also interesting in term of reducing the design effect close to 1, probably by spreading the sample in each community and hence, increasing the heterogeneity in each cluster.

A higher decrease in GAM prevalence (0.26 % per visit) was observed in LP compared to cross-sectional surveys. It is probable that in the field, information provided to families by our staff and referral of malnourished children have improved the nutritional status of the children within these sentinel villages. Likewise, the observational bias reported in a recent study piloted in Northern Nigeria by Médecins sans Frontières (MSF) was a progressive deviation of the nutritional status of the sentinel site from that of the wider community it is presumed to represent [10]. In the MSF study, the prevalence of GAM decreased by 1.6 % (95 % CI 0.4–2.7 %; p = 0.012) relative to the prevalence observed during the previous visit. The smaller observational bias in our study might be partially explained by the normal–normal conjugate analysis that uses the same prior for both data sources (see limitations). Other explanations might include program coverage as well as impact of messages and referral in these two contexts. The quality of the surveillance system improved over time which might explain why LP and cross-sectional survey estimates are more different at the beginning (i.e. March 2011) than in 2013. Globally we conclude that in the course of the LP surveys, there was a slight observational bias (defined as the difference in GAM prevalence between the two data sources) that would probably become statistically significant after a 3 year and 10 months period. It would seem reasonable to take a new sample of sentinel sites every 3 years.

The thresholds for GAM by MUAC are internationally agreed and apply to any child in the 6–59 months of age group. However, as surveillance systems do not focus on single estimates but rather look at trends, there were difficulties in interpreting the curves during the first year, where no comparison was available. For the following years, the historical limit approach was applied. However, there is no clear guidance on how to use this approach in the nutrition sector, as it is more commonly used for infectious diseases [31].

While the system is still ongoing and has even been extended to Gnagna province, questions remain regarding its sustainability. The same system was set up in Montserrado, Liberia, and was stopped after 21 months. Reasons for stopping were: (1) low prevalence of GAM; (2) weak connection with ACF programs; (3) no possible integration of this methodology into the national system; (4) the challenging issue of urban sampling and the fact that few data were available to assess the validity of our results in greater Monrovia. In Burkina Faso, the Ministry of Agriculture (MoA) was first interested to use this methodology to develop an early warning system (EWS) for food and nutrition. However, its capacities remain limited and there is actually no fund for a EWR. Furthermore, they are still doubts that MoA is responsible to collect nutritional data. Alternatively, the system could be extended to other areas through other partners. Suggestions to improve the uptake include more regular workshops, set up of a dedicated website and a better design of the newsletter.

The LP system provides monthly information that allows prompt interventions if needed. Alternative methodologies, such as cross-sectional surveys, are usually not frequent enough for early warning systems. Furthermore, cost of the LP system is limited with around 50,000 dollars per year for the 3 LHZ. In comparison, costs of cross-sectional surveys are around 20,000 dollars per survey. Finally, its set up is easier than repeated cross-sectional surveys. We think the LP methodology is adapted to areas with high burden of acute undernutrition and poor access to feeding centres that require continuous monitoring and an early warning system.

There were some limitations in our study. It would have been better if the number and timing of the cross-sectional surveys were the same as surveillance rounds. It would have allowed us to better compare trends from LP and repeated-cross sectional data over shorter time periods. Furthermore, in order to compare with cross-sectional surveys’ area, we had to pool 3 LHZs, increasing the sample size. This has limited our ability to validate MUAC trends for each LHZ. However, it has improved the precision of the LP estimates, increasing our power. The use of the Bayesian normal–normal conjugate analysis may have biased the posterior estimate towards the prior mode, which was the same for the surveillance data and the cross-sectional survey data. We use the same prior for all applications. The prior was, however, tested with computer based simulations using data derived from cross-sectional surveys; we found that it did not introduce a bias even when used with relatively small likelihood survey sample sizes such as n = 96 and n = 132. Recent literature [17, 18] has identified small systematic biases in PROBIT estimates in given populations. This is problematic when estimating prevalence in one-shot cross-sectional surveys. Systematic bias is less important for surveillance systems. Surveillance systems accept bias and attempt to keep it systematic (i.e. consistent) over time in order to observe patterns of change. To detect change, precision (reliability, repeatability) is a more important issue than accuracy (bias) in surveillance applications. For a given sample size, the PROBIT method has greater precision than the more “conventional” methods [17, 18]. The Bayesian approach contributes some additional information (i.e. the prior) which also improves precision. The cohort approach also reduces between-round sampling variation. The use of the Bayesian PROBIT estimator and an open cohort in the LP method reflects the importance of precision in surveillance applications. Other methods, such as conventional prevalence method, can be used to analyze the data as a 6 × 22 cluster sample. They won’t, however, provide the same precision as the Bayesian PROBIT estimator.

Finally, our study was not designed and did not aim to assess the Bayesian-PROBIT methodology in estimating GAM prevalence in comparison to SMART survey methodology. Further studies may be needed to validate this methodology in estimating GAM prevalence in different contexts.

## Conclusions

This paper presented a methodology for nutrition surveillance using a small open cohort from community-based sentinel sites in a rural setting of Burkina Faso. This methodology has proved to be valid in following trends of GAM prevalence over a 3 years period without selection bias; conversely, a slight but significant observational bias was detected, requiring periodical reselection of sentinel sites. We recommend this approach for areas with high burden of acute under-nutrition and poor access to feeding centres where early warning systems are strongly needed. Advocacy is necessary to develop sustainable nutrition surveillance system and to support the use of surveillance data in guiding nutritional programs.
